# Effects of Knowledge, Attitudes, and Practices of Primary Care Providers on Antibiotic Selection, United States

**DOI:** 10.3201/eid2012.140331

**Published:** 2014-12

**Authors:** Guillermo V. Sanchez, Rebecca M. Roberts, Alison P. Albert, Darcia D. Johnson, Lauri A. Hicks

**Affiliations:** Centers for Disease Control and Prevention, Atlanta, Georgia, USA

**Keywords:** Antibacterial drugs, resistance, antimicrobial, antibiotics, prescribing, qualitative research, interviews, community, physician assistant, nurse practitioner, respiratory tract infections, primary care provider, PCP, United States

## Abstract

Appropriate selection of antibiotic drugs is critical to optimize treatment of infections and limit the spread of antibiotic resistance. To better inform public health efforts to improve prescribing of antibiotic drugs, we conducted in-depth interviews with 36 primary care providers in the United States (physicians, nurse practitioners, and physician assistants) to explore knowledge, attitudes, and self-reported practices regarding antibiotic drug resistance and antibiotic drug selection for common infections. Participants were generally familiar with guideline recommendations for antibiotic drug selection for common infections, but did not always comply with them. Reasons for nonadherence included the belief that nonrecommended agents are more likely to cure an infection, concern for patient or parent satisfaction, and fear of infectious complications. Providers inconsistently defined broad- and narrow-spectrum antibiotic agents. There was widespread concern for antibiotic resistance; however, it was not commonly considered when selecting therapy. Strategies to encourage use of first-line agents are needed in addition to limiting unnecessary prescribing of antibiotic drugs.

## Abstract 2

Primary care providers were familiar with recommendations for antibiotic drug selection for common infections, but do not always comply with them.

Antibiotic prescribing guidelines establish standards of care, help focus efforts on quality improvement, and have been shown to improve patient outcomes (*1*–*3*). Many guidelines emphasize the importance of diagnostic certainty for the management of bacterial upper respiratory tract infections and promote β-lactam agents, such as amoxicillin or amoxicillin-clavulanate, as the preferred first-line therapy (*4*–*6*). Studies indicate that health care providers often do not adhere to established clinical practice guidelines for the management of common infections (*7*–*9*). Prescribing rates for second-line, broad-spectrum antibiotics among outpatients have increased, contributing to a growing problem of antibiotic-resistant infections (*10*–*14*). It is not clear whether nonadherence is related to lack of familiarity with clinical practice guidelines or if other factors influence antibiotic selection once a diagnosis is established.

Published qualitative studies that have examined antibiotic selection among primary care providers (PCPs) are outdated and focus on non-US–based physicians; they do not include nurse practitioners or physician assistants, who together comprise >25% of the US primary care workforce (*15*–*22*). The objectives of this study are to explore US PCP knowledge, attitudes, and self-reported practices (KAPs) concerning antibiotic therapy, assess factors that influence provider antibiotic choice, and provide an update on PCP attitudes regarding antibiotic resistance.

## Methods

We conducted in-depth interviews by digitally recorded telephone calls, and transcribed the recordings to text to accurately and reliably assess PCP KAPs. The qualitative method of an open-ended interview by telephone was chosen to ensure candid and truthful answers from participants. We composed a screening questionnaire to recruit physicians, nurse practitioners (NPs), and physician assistants (PAs) from a nationwide marketing database in the United States. We initially contacted and screened potential participants (Technical Appendix Section A) using telephones, email, and fax transmission of documents. Inclusion criteria included self-reporting of spending >50% of medical practice time in direct patient contact in a primary care setting, >30 years of age, and fluency in the English language. Interviewees were excluded from this study if they had an immediate family member who was employed in an industry that could represent a conflict of interest, including advertising or public relations, the federal government, market research, news media, or the pharmaceutical industry; if they had board certification in a subspecialty outside of primary care; or if they had practiced medicine >30 years at the time of recruitment.

Thirty-six PCPs were selected for the study. Specialties for the 27 physician participants selected included pediatrics (n = 9), family medicine (n = 9), and internal medicine (n = 9). Among PAs (n = 4) and NPs (n = 5), 6 practiced in family medicine setting and 3 practiced in pediatrics. Provider specialty, years in practice, and demographic information are described in Table 1.

**Table 1:** Characteristisc of primary care providers interviewed for knowledge, attitudes, and practices in antibiotic drug selection, United States*

Characteristic	Physician, n = 27	NP or PA, n = 9
Sex		
M	18	1
F	9	8
Race/ethnicity		
White	18	9
Black	4	0
Asian	3	0
Hispanic	1	0
Other	1	0
Years in practice		
<10	5	5
10–20	11	3
21–30	11	1
Medical specialty		
Pediatrics	9	3
Family medicine	9	6
Internal medicine	9	0

*NP, nurse practitioner; PA, physician assistant.

Interviews were conducted during May 2013. One professional moderator who had >25 years of moderating experience conducted all interviews. Based on a discussion guide prepared by our research team (data not shown), participants were first informed of the sponsoring organization (Centers for Disease Control and Prevention, Atlanta, GA, USA), the planned use of data, and presence of listeners; they were then asked “warm-up” questions about the practice setting in which the participant worked and the patient populations whom they served. Next, each interview proceeded through an ordered list of open-ended questions on self-reported antibiotic prescribing practices, perceived prescribing practices of their peers, attitudes toward clinical practice guidelines for common bacterial infections, knowledge of narrow- versus broad-spectrum antibiotic agents, preferred resources and methods for medical education and antibiotic treatment, and attitudes toward antibiotic resistance.

Participants were provided a worksheet before the interview (Technical Appendix, Section B) which asked them to rank each of 12 factors on the basis of its perceived influence on antibiotic selection when an antibiotic is indicated (e.g., illness severity, patient demand for an antibiotic, or clinical practice guidelines). Worksheet answers were discussed and recorded during the interview. 

To assess compliance with clinical practice guidelines and to evaluate clinical decision-making, each participant was given a clinical vignette about a patient (Technical Appendix, Section C) who had a diagnosis of an acute bacterial infection: acute otitis media (AOM) for pediatricians, acute bacterial rhinosinusitis for internists, acute uncomplicated cystitis for family practitioners, and group A streptococcal pharyngitis for PAs and NPs. The participant was asked to explain his or her rationale in choosing an antibiotic agent and why other PCPs might choose nonrecommended antibiotics. For the purposes of this study, antibiotics considered to be broad-spectrum include penicillins containing β-lactamase inhibitors (e.g., amoxicillin/clavulanate), second through fifth generation cephalosporins, macrolides, quinolones, and lincomycin derivatives. Narrow-spectrum agents include penicillins (e.g., amoxicillin), first generation cephalosporins, sulfonamides, and nitrofurantoin. Each participant received a cash incentive after the interview in exchange for participating.

Interviews were transcribed by project staff, and each member of the research team either listened to interview recordings or read corresponding interview transcripts. Relevant excerpts were coded into compilations of references to specific themes and were used to identify the most frequent responses to discussion topics outlined in the discussion guide. We identified common themes using both inductive and deductive methods that were reviewed by all authors. Author disagreement on theme selections were discussed until a consensus was met. If no consensus was met by most study authors, the theme was excluded from our results. We performed in-depth analyses of themes by reading and coding transcribed responses using Nvivo 9 (QSR International, Burlington, MA). This study was reviewed and approved for exemption status by the Human Research Protection Office of the Centers for Disease Control and Prevention.

## Results

We conducted 36 interviews, each lasting ≈45 minutes. Through analysis of provider in-depth interviews, several common themes regarding antibiotic prescribing and antibiotic resistance were identified (Table 2).

**Table 2:** Topics and quotations from in-depth interviews with primary care providers regarding antibiotic therapy and antibiotic resistance, United States

Topic	Quotation
Antibiotic selection	*“We as doctors are business people. We’re no different than running a shoe store. If somebody comes in and wants black shoes, you don’t sell them white shoes. And if you do, they get upset. You can convince a patient, look if I were you I wouldn’t take this antibiotic… but patients in general don’t understand that concept of not taking it if you don’t need it… [and] if you don’t give it to them, they don’t come back to you.”*
	*“The patient [may] call you up and… tell you … call me in some X, Y or Z because everybody wants to hurry up and get better faster. Sometimes [you worry] about 1-800 call you-know-lawyer. [Maybe] you don’t have a standing relationship with the patient and you don’t know [if they will] come back if they are not getting better. All of those things [affect antibiotic selection]. But if you are pretty comfortable and have a good relationship [with a patient], you [may] not necessarily go straight to a broad-spectrum antibiotic.”*
	*“[Broad-spectrum antibiotics] take the thinking out of it for me so that I am not trying to figure out what the organism is and [which] particular antibiotic treats the organism.”*
	*“It’s very simple. Patients come to the doctor and they want an antibiotic. If they don’t get better, they get upset… I had a patient who came to me who had bronchitis and I started her on azithromycin… however, she did not get better. She came to see my colleague and [he] did not change the antibiotic, but gave her Prednisone and that got her better within 24 hours. She was mad at me, because I apparently did not give her ‘the right antibiotic’ and my partner did.”*
Broad- and narrow-spectrum definitions	*“If it’s narrow, it [covers] one particular class of organism like gram-negative. If it’s broad, it’s going to be different types like gram-negative, gram-positive, anaerobes to treat a wider spectrum of infections.”*
	*“The more bacteria the antibiotic works …against, you call it broader. If this antibiotic only works against one or two types of bacteria, then that is a narrow-spectrum [antibiotic].”*
	*“The one thing no one’s going to argue about is penicillin being narrow-spectrum.”*
	*“Amoxicillin is a great example of a very broad-spectrum antibiotic.”*
	*“I guess it is pretty subjective, the definition [of broad- versus narrow-spectrum antibiotics].”*
Education and resources for antibiotic treatment	*“During residency…when you see patients with different conditions and decide how you want to treat them and see how the attending… chooses to treat them, I think that's when you learn the most.”*
	*“I think there needs to be more education to providers about the real dangers of antibiotic resistance. You get taught that in school and … you don't ever hear about it again, and you get busy in your practice, it's not your number one priority. And you… get to where you really don't think about it, but it is a huge issue.”*
	*“I think [focusing education efforts only on students and residents] would be a mistake. I think that’s the conclusion you would draw if you assume the rest of the system stayed unchanged. If you just focus on the young doctors you're still going to wait 15 years for that change to take place.”*
Changing prescribing habits	*“Habits are hard to change. I will say that. So if someone’s used to writing Zithromax… they’re not going to stop because it’s easy, patients want it, and they want the patients to go away and be happy. But I think pushing the knowledge is helpful, and change takes time.”*
	*“[Physicians] tend to be rigid. That’s it, really; a new generation that has learned its own rigid rules that come in and replace the previous… It’s not that any new crop of doctors is any more malleable, it’s the fact that everybody’s not malleable… [they] resort to the way they were during residency.”*
	*“Patients don’t realize [antibiotic resistance could affect them] and some of them don’t even care. You can say ‘one day you’re going to [have a resistant infection]’ and they’ll say ‘oh, they’ll make more [drugs]’.”*
Antibiotic resistance	*“It is very scary because we are seeing increasingly resistant germs. We had a lady in the office the other day [with] a urinary infection [resistant to] every single oral antibiotic on the entire list of the culture and sensitivity report. There wasn’t a single oral medicine that could be used... It was resistant to everything.”*
	*“We haven’t had a new antibiotic for at least 10 years and something is going to happen one of these days. We are going to get a big, big multi-drug resistant bacteria and we are going to end up with nothing to treat this thing… and then we are going to be in trouble. That is my main concern.”*
	*“I had a patient with an abscess on his nose, and [a dermatologist] agreed with the Bactrim I started, I see the patient today, looks 100% better, but needs to stay on the antibiotics. “Well, I want to stop,” [the patient said]. Why? “I’m worried about antibiotic resistance,” which is a fair assessment.”*

### Antibiotic Selection

PCPs generally cited little difficulty selecting antibiotic treatments for common infections, but indicated that allergies, complicated medical histories, and recurrent infections regularly make antibiotic selection more challenging. Previous experience and familiarity with an agent were frequently cited as influential factors when choosing antibiotic therapy. Results from the ranking exercise suggest that illness severity, medical history, and clinical practice guidelines were important considerations across all specialties.

When asked why their peers made inappropriate choices regarding antibiotic selection, responses varied. The most common perceived reasons for inappropriate antibiotic prescribing were pressure from patients or parents; desire to prevent litigation as a result of complications of infections; concern for patient or parent satisfaction with the visit; and perceived decreases in visit length. Illnesses for which participants believed appropriate antibiotic selection was more difficult for their peers included sinus infections and recurrent urinary tract infections.

Broad-spectrum agents were widely thought to be more successful for curing an infection than narrow-spectrum antibiotics. One participant noted that broad-spectrum antibiotics are often chosen because less uncertainty exists regarding the adequacy of antimicrobial activity, which is why they may be commonly selected even if a narrow-spectrum agent is indicated. When asked whether antibiotic spectrum was a consideration when prescribing an antibiotic, participants indicated that it was generally not as important as choosing a drug known to successfully treat the infection. Choosing a narrow-spectrum agent was perceived to be a more attractive option when the diagnosis was more certain or when a patient was perceived to have a benign clinical condition.

PCPs commonly perceived that patients expect an antibiotic for clinical visits, contributing to a shared feeling of pressure among PCPs to satisfy patients. Concern was further expressed that patient satisfaction scores are a common method by which providers are evaluated by hospital administration and insurance companies and that this may inadvertently contribute to more frequent antibiotic prescribing. Some participants believed that such evaluation methods could be a barrier to appropriate antibiotic use. PCPs in private practice said they were particularly sensitive to patient desires and were concerned that their patients might leave their practice in favor of another physician if their expectations were not met.

Several participants believed other health care providers’ prescribing behaviors negatively affected their own. For example, if a patient sees a PCP and receives a course of antibiotics, and later visits another PCP for a separate infection with the same symptoms, they have the established expectation to receive another course of antibiotics. As a result, the new provider feels pressured by such expectations to prescribe antibiotics even if they may be inappropriate.

Responses to the clinical vignettes were generally in line with the recommended first-line therapies. Among 9 participants given a case describing acute bacterial sinusitis, 7 appropriately chose amoxicillin or amoxicillin/clavulanate. One participant chose to treat the patient with moxifloxacin, and another chose azithromycin. Sixteen of 18 PCPs given clinical vignettes of either AOM or group A streptococcal pharyngitis selected the recommended first-line treatments amoxicillin or penicillin. In response to the clinical vignette for acute cystitis, only 4 of 9 family practitioners selected the first-line agent nitrofurantoin; 4 others chose to treat the patient with a fluoroquinolone, and 1 chose to prescribe trimethoprim-sulfamethoxazole.

### Broad- and Narrow-Spectrum Definitions

Participants had no shared definition of “broad-spectrum antibiotic” or “narrow-spectrum antibiotic” regardless of specialty or number of years in practice. When asked to define broad- versus narrow-spectrum antibiotics, responses varied (Table 2). Although many participants believed antibiotic spectrum referred to the classes of bacteria (gram-positive, gram-negative, or anaerobic) treated, others believed it referred to the number of pathogens addressed (e.g., a narrow-spectrum antibiotic might affect only 2 or 3 pathogens and a broad-spectrum antibiotic might affect many).

Although some participants correctly identified amoxicillin as a narrow-spectrum agent, and azithromycin as a broad-spectrum agent, many participants were uncertain of the spectrum of antimicrobial activity for these 2 widely used antibiotics. In general, participants were able to correctly identify fluoroquinolones and later-generation cephalosporins as broad-spectrum agents, especially in relation to other antibiotics. Many participants agreed that broad- and narrow-spectrum antibiotics were not uniformly defined among their colleagues.

### Changing Prescribing Habits

Respondents believed that changing prescribing behaviors is a difficult task. When asked why some health care providers are reluctant to change their prescribing practices, many participants believed that health care providers are used to the way they have been practicing medicine for years. They believed that even when providers are familiar with established clinical practice guidelines, it may not matter because they seldom change their prescribing behaviors. Several respondents believed the best way to change antibiotic prescribing behavior is to change the expectations of patients so the pressure to prescribe antibiotics is reduced.

Respondents’ preference of how they would like to receive information regarding antibiotic selection varied. Some participants suggested incorporating information in the electronic medical record to improve prescribing (e.g., a clinical decision support prompt would display on the computer in use if a physician entered an order for a prescription that is not standard for the diagnosis). One frequent suggestion to improve antibiotic prescribing was to have a quick reference guide for each major diagnosis, including antibiotic indications and causative pathogens. Another suggestion was to have a mobile telephone application available without Internet access. Several respondents saw value in having easy access to antibiotic resistance data for their local area.

### Concern for Antibiotic Resistance

Participants generally agreed that antibiotic resistance is a concern for their patients and for public health; however, it was not commonly mentioned as a factor when considering which antibiotic to prescribe. Several participants expressed serious concerns about the availability of effective antibiotic therapies in the future related to increases in antibiotic resistance. Respondents also noted an increasing frequency of patients who were reluctant to begin antibiotics or complete their course of antibiotics because of concerns about antibiotic resistance.

## Discussion

Our study shows that PCPs do not always adhere to guidelines because they believe broad-spectrum antibiotics may be more likely to cure an infection, a finding corroborated by the well-documented overuse of broad-spectrum antibiotics (*23*). However, the perception of better cure rates when using broad-spectrum agents is unfounded. For example, *Streptococcus pneumoniae,* a pathogen frequently implicated in bacterial respiratory infections, has a much higher prevalence of resistance to macrolides than it does to amoxicillin (*24*,*25*). Nevertheless, azithromycin is often chosen over amoxicillin for the empiric treatment for AOM, sinusitis, and other respiratory infections (*25*,*26*). Similarly, for the treatment for acute uncomplicated cystitis, widespread use of fluoroquinolones has contributed to rising antimicrobial resistance, rendering ciprofloxacin far less active against urinary tract *Escherichia coli* infections than the more narrow-spectrum, first-line agent nitrofurantoin (*27*). The perceived association between broad-spectrum antibiotic use and better cure rates may regularly contribute to inappropriate antibiotic selection and warrants further attention from appropriate antibiotic use initiatives.

There is no widely accepted definition of broad- versus narrow-spectrum antibiotics among PCPs or their professional organizations. Although a list of “antibiotics of concern” has been published by the National Committee on Quality Assurance (*28*) and has been used in previous research to classify antibiotics as broad-spectrum (*29*,*30*), the list was not originally intended for this purpose. Clinical practice guidelines emphasize use of narrow-spectrum antimicrobial agents instead of their broad-spectrum counterparts (*4*,*5*,*31*,*32*). However, the effect of these messages may be limited because of lack of clarity regarding what constitutes a narrow- versus broad-spectrum antibiotic. For example, few participants in our study were able to confidently categorize macrolides and penicillins, which are among the most commonly prescribed classes of antibiotics (*33*), as broad or narrow spectrum. Although this issue is largely one of semantics, it has critical implications for medical education, public health messaging, and community antibiotic resistance. Communication to PCPs related to antibiotic choice should not focus on dichotomous narrow- versus broad-spectrum terminology, but rather promote specific recommended first-line and targeted antibiotic therapies for individual diagnoses.

Compared with results of previous qualitative studies, PCPs participating in this study expressed greater urgency regarding antibiotic resistance. For example, in a 1998 qualitative study exploring driving factors of antibiotic misuse, a principal barrier to change in antibiotic prescribing was the attitude that antibiotic resistance was not an important problem (*19*). Another study published in the same year noted similar findings (*21*). Conversely, not a single provider in this study dismissed antibiotic resistance as being a minor issue, and several expressed grave concerns about antibiotic resistance based on their own experiences.

Modifying prescriber behavior is a complex and difficult task. Multifaceted interventions that involve a combination of interactive group meetings, outreach visits to individual physicians, physician reminders, or patient-based interventions (e.g., delayed prescribing practices) have shown the most promise in changing prescribing behaviors in ambulatory care settings (*34*,*35*). Previous studies confirm that patients desire antibiotics less frequently than providers perceive and that inappropriate prescribing is a common result of this miscommunication (*21*,*36*,*37*). This finding suggests that an effective target for intervention is narrowing the gap between patient expectations and clinician perception of these expectations for antibiotics. Regardless of the intervention considered for promoting appropriate antibiotic use, the concerns of PCPs highlighted in this study should be addressed. This includes reassuring providers of the efficacy of first-line and targeted therapies, clarifying the role of antibiotic prescriptions in patient satisfaction, and providing resources that streamline patient education efforts in primary care settings.

This study has limitations, however. Although in-depth interviews are an effective method to explore individual providers’ KAPs, we cannot generalize our findings to the PCP population as a whole because of the lack of external validity inherent in this type of qualitative research. Similarly, the clinicians who were screened, selected for participation, and agreed to be interviewed may hold stronger opinions about this topic than those who were excluded or declined to participate.

Our study suggests that inappropriate antibiotic selection among PCPs is not caused by lack of familiarity with guideline recommendations. Rather, this practice is the result of a complex interaction between perceived better cure rates for nonrecommended therapies and attempts to meet demands typical in primary care settings.

Future research efforts should be aimed at investigating effective incentives for appropriate antibiotic prescribing and determining alternative communication strategies to encourage use of first-line agents. Although most efforts have focused on reducing unnecessary antibiotic use, more research is needed to clarify which interventions improve antibiotic selection. Although awareness regarding antibiotic resistance appears to be improving, ongoing education efforts promoting appropriate antibiotic use among both patients and health care providers (e.g., CDC’s Get Smart: Know When Antibiotics Work Program) are critical to addressing the growing threat of antibiotic resistance.

Technical Appendix Worksheet and case scenario interview for primary care providers participating in the study on antibiotic selection and screening instrument used to select participants.

## References

[1] Asadi L, Eurich DT, Gamble JM, Minhas-Sandhu JK, Marrie TJ, Majumdar SR. Impact of guideline-concordant antibiotics and macrolide/beta-lactam combinations in 3203 patients hospitalized with pneumonia: prospective cohort study. Clin Microbiol Infect. 2013;19:257–64. 10.1111/j.1469-0691.2012.03783.x 22404691

[2] Asadi L, Eurich DT, Gamble JM, Minhas-Sandhu JK, Marrie TJ, Majumdar SR. Guideline adherence and macrolides reduced mortality in outpatients with pneumonia. Respir Med. 2012;106:451–8 . 10.1016/j.rmed.2011.11.017 22182341

[3] Garber AM. Evidence-based guidelines as a foundation for performance incentives. Health Aff. 2005;24:174–9. 10.1377/hlthaff.24.1.174 15647228

[4] Chow AW, Benninger MS, Brook I, Brozek JL, Goldstein EJ, Hicks LA, IDSA clinical practice guideline for acute bacterial rhinosinusitis in children and adults. Clin Infect Dis. 2012;54:e72–112. 10.1093/cid/cis370 22438350

[5] Lieberthal AS, Carroll AE, Chonmaitree T, Ganiats TG, Hoberman A, Jackson MA, The diagnosis and management of acute otitis media. Pediatrics. 2013;131:e964–99. 10.1542/peds.2012-3488 23439909

[6] Shulman ST, Bisno AL, Clegg HW, Gerber MA, Kaplan EL, Lee G, Clinical practice guideline for the diagnosis and management of group A streptococcal pharyngitis: 2012 update by the Infectious Diseases Society of America. Clin Infect Dis. 2012;55:1279–82. 10.1093/cid/cis847 23091044

[7] Hong SY, Taur Y, Jordan MR, Wanke C. Antimicrobial prescribing in the USA for adult acute pharyngitis in relation to treatment guidelines. J Eval Clin Pract. 2011;17:1176–83. 10.1111/j.1365-2753.2010.01495.x 20586844 PMC2978269

[8] Barnett ML, Linder JA. Antibiotic prescribing to adults with sore throat in the United States, 1997–2010. JAMA Intern Med. 2014;174:138–40. 10.1001/jamainternmed.2013.11673 24091806 PMC4526245

[9] Crocker A, Alweis R, Scheirer J, Schamel S, Wasser T, Levingood K. Factors affecting adherence to evidence-based guidelines in the treatment of URI, sinusitis, and pharyngitis. J Community Hosp Intern Med Perspect. 2013;3. 10.3402/jchimp.v3i2.20744 PMC3716032 23882403

[10] Steinman MA, Gonzales R, Linder JA, Landefeld CS. Changing use of antibiotics in community-based outpatient practice, 1991–1999. Ann Intern Med. 2003;138:525–33. 10.7326/0003-4819-138-7-200304010-00008 12667022

[11] Linder JA, Huang ES, Steinman MA, Gonzales R, Stafford RS. Fluoroquinolone prescribing in the United States: 1995 to 2002. Am J Med. 2005;118:259–68. 10.1016/j.amjmed.2004.09.015 15745724

[12] Shapiro DJ, Hicks LA, Pavia AT, Hersh AL. Antibiotic prescribing for adults in ambulatory care in the USA, 2007–09. J Antimicrob Chemother. Epub 2013 Jul 25. 10.1093/jac/dkt301 23887867

[13] Steinman MA, Landefeld CS, Gonzales R. Predictors of broad-spectrum antibiotic prescribing for acute respiratory tract infections in adult primary care. JAMA. 2003;289:719–25. 10.1001/jama.289.6.719 12585950

[14] Shehab N, Patel PR, Srinivasan A, Budnitz DS. Emergency department visits for antibiotic-associated adverse events. Clin Infect Dis. 2008;47:735–43. 10.1086/591126 18694344

[15] Cadieux G, Tamblyn R, Dauphinee D, Libman M. Predictors of inappropriate antibiotic prescribing among primary care physicians. CMAJ. 2007;177:877–83. 10.1503/cmaj.070151 17923655 PMC1995156

[16] Björkman I, Erntell M, Roing M, Lundborg CS. Infectious disease management in primary care: perceptions of GPs. BMC Fam Pract. 2011;12:1. 10.1186/1471-2296-12-1 21223592 PMC3025850

[17] Tonkin-Crine S, Yardley L, Coenen S, Fernandez-Vandellos P, Krawczyk J, Touboul P, GPs' views in five European countries of interventions to promote prudent antibiotic use. Br J Gen Pract. 2011;61:e252–61. 10.3399/bjgp11X572445 21619749 PMC3080230

[18] Linder JA, Schnipper JL, Tsurikova R, Volk LA, Middleton B. Self-reported familiarity with acute respiratory infection guidelines and antibiotic prescribing in primary care. Int J Qual Health Care. 2010;22:469–75. 10.1093/intqhc/mzq052 20935008 PMC3003551

[19] Barden LS, Dowell SF, Schwartz B, Lackey C. Current attitudes regarding use of antimicrobial agents: results from physician's and parents' focus group discussions. Clin Pediatr. 1998;37:665–71. 10.1177/000992289803701104 9825210

[20] Wigton RS, Darr CA, Corbett KK, Nickol DR, Gonzales R. How do community practitioners decide whether to prescribe antibiotics for acute respiratory tract infections? J Gen Intern Med. 2008;23:1615–20. 10.1007/s11606-008-0707-9 18622651 PMC2533389

[21] Butler CC, Rollnick S, Pill R, Maggs-Rapport F, Stott N. Understanding the culture of prescribing: qualitative study of general practitioners' and patients' perceptions of antibiotics for sore throats. BMJ. 1998;317:637–42. 10.1136/bmj.317.7159.637 9727992 PMC28658

[22] Agency for Healthcare Research and Quality. Primary care workforce and stats: overview. Rockville, MD: U.S. Department of Health and Human Services; January 2012 [cited 3Nov 1]. http://www.ahrq.gov/research/findings/factsheets/primary/pcworkforce/index.html

[23] Shapiro DJ, Hicks LA, Pavia AT, Hersh AL. Antibiotic prescribing for adults in ambulatory care in the USA, 2007–09. J Antimicrob Chemother. 2014;69:234–40. 10.1093/jac/dkt301 23887867

[24] Hicks LA, Chien YW, Taylor TH Jr, Haber M, Klugman KP, Active Bacterial Core Surveillance T. Outpatient antibiotic prescribing and nonsusceptible *Streptococcus pneumoniae* in the United States, 1996–2003. Clin Infect Dis. 2011;53:631–9. 10.1093/cid/cir443 21890767

[25] Courter JD, Baker WL, Nowak KS, Smogowicz LA, Desjardins LL, Coleman CI, Increased clinical failures when treating acute otitis media with macrolides: a meta-analysis. Ann Pharmacother. 2010;44:471–8. 10.1345/aph.1M344 20150506

[26] Fairlie T, Shapiro DJ, Hersh AL, Hicks LA. National trends in visit rates and antibiotic prescribing for adults with acute sinusitis. Arch Intern Med. 2012;172:1513–4. 10.1001/archinternmed.2012.4089 23007315

[27] Sanchez GV, Master RN, Karlowsky JA, Bordon JM. In vitro antimicrobial resistance of urinary *Escherichia co*li isolates among U.S. outpatients from 2000 to 2010. Antimicrob Agents Chemother. 2012;56:2181–3. 10.1128/AAC.06060-11 22252813 PMC3318377

[28] Healthcare Effectiveness Data and Information Set. (HEDros Inf Serv.) & Quality Measurement: HEDros Inf Serv. 2014 National Drug Code List [cited 2014 June 23]. http://www.ncqa.org/HEDISQualityMeasurement/HEDISMeasures/HEDIS2014/HEDIS2014FinalNDCLists.aspx

[29] Lee GC, Reveles KR, Attridge RT, Lawson KA, Mansi IA, Lewis JS II, Outpatient antibiotic prescribing in the United States: 2000 to 2010. BMC Med. 2014;12:96. 10.1186/1741-7015-12-96 24916809 PMC4066694

[30] Steinman MA, Yang KY, Byron SC, Maselli JH, Gonzales R. Variation in outpatient antibiotic prescribing in the United States. Am J Manag Care. 2009;15:861–8. 20001167 PMC2910590

[31] Gupta K, Hooton TM, Naber KG, Wullt B, Colgan R, Miller LG, International clinical practice guidelines for the treatment of acute uncomplicated cystitis and pyelonephritis in women: A 2010 update by the Infectious Diseases Society of America and the European Society for Microbiology and Infectious Diseases. Clin Infect Dis. 2011;52:e103–20. 10.1093/cid/ciq257 21292654

[32] Mandell LA, Wunderink RG, Anzueto A, Bartlett JG, Campbell GD, Dean NC, Infectious Diseases Society of America/American Thoracic Society consensus guidelines on the management of community-acquired pneumonia in adults. Clin Infect Dis. 2007;44(Suppl 2):S27–72. 10.1086/511159 17278083 PMC7107997

[33] Hicks LA, Taylor TH Jr, Hunkler RJUS. outpatient antibiotic prescribing, 2010. N Engl J Med. 2013;368:1461–2. 10.1056/NEJMc1212055 23574140

[34] Arnold SR, Straus SE. Interventions to improve antibiotic prescribing practices in ambulatory care. Cochrane Database Syst Rev. 2005;19. 16235325 10.1002/14651858.CD003539.pub2 PMC7003679

[35] Weber JT. Antimicrobial resistance: beyond the breakpoint. Basel (Switzerland): Karger Books; 2010.

[36] Stivers T, Mangione-Smith R, Elliott MN, McDonald L, Heritage J. Why do physicians think parents expect antibiotics? What parents report vs what physicians believe. J Fam Pract. 2003;52:140–8. 12585992

[37] Vanden Eng J, Marcus R, Hadler JL, Imhoff B, Vugia DJ, Cieslak PR, Consumer attitudes and use of antibiotics. Emerg Infect Dis. 2003;9:1128–35. 10.3201/eid0909.020591 14519251 PMC3016767

