# Supplementary material for: Effects of Knowledge, Attitudes, and Practices of Primary Care Providers on Antibiotic Selection, United States
Source: Emerg Infect Dis. 2014 Dec;20(12):2041–7. doi: 10.3201/eid2012.140331 (PMC4257826; doi:10.3201/eid2012.140331)
Supplement: Technical Appendix — Worksheet and case scenario interview for primary care providers participating in the study on antibiotic selection and screening instrument used to select participants. [file 14-0331-Techapp-s1.pdf]

DOI: http://dx.doi.org/10.3201/eid2012.1340331

Knowledge, Attitudes, and Practices used by Primary Care Providers in Antibiotic Drug Selection, United States

Technical Appendix: Tools Used to Interview Study Participants

Technical Appendix Section A

Screening Instrument

Antibiotic Selection — Family Practice In-depth Interviews

Hello, my name is ____________________with _____________ a market research firm. We are talking today with physicians in the area about a public health issue. We are not selling anything. We have a few brief questions and if you qualify and are interested, we will invite you to take part in an interview that will take place at a later date. To see if you qualify for an interview, I need to ask you a few questions.

1. Is anyone in your immediate family employed in the following industries?

   [IF YES TO ANY, THANK AND TERMINATE]
   01 Advertising or public relations
   02 Market research
   03 News media – works for a newspaper, TV station, radio station or some other form of news media
   04 Federal government
   05 Pharmaceutical company
2. Which, if any, of the following describe your specialty?
   01 Pediatrician **[RECRUIT FOR PEDIATRIC IDIs*]**
   02 Family Medicine
   03 Internal Medicine **[RECRUIT FOR INTERNISTS IDIs]**
   04 Other **[THANK AND TERMINATE]**
   [DOCUMENT ON GRID]
3. Are you board-certified in family medicine?
   01 Yes
   02 No **[THANK AND TERMINATE]**
4. Are you board-certified in any other specialty?
   01 Yes **[THANK AND TERMINATE]**
   02 No
5. About what percentage of your time do you spend in direct contact with patients?
   01 50% or more
   02 Less than 50% **[THANK AND TERMINATE]**

6. How many years have you been in practice?
   01 Fewer than ten years
   02 10 to 20 years
   03 21 to 30 years
   04 31 years or more **[THANK AND TERMINATE]**
   [DOCUMENT ON GRID]
7. Is your primary work
   01 In a primary care setting **[RECRUIT AT LEAST 4/MARKET]**
   02 Other
   [THANK AND TERMINATE]
8. What is the business name of your practice or clinic?
   ____
   [NO MORE THAN 1 RESPONDENT FROM A PRACTICE OR CLINIC]
   [DO NOT RECORD NAME ON GRID]
9. Would you please tell me your race?
   01 White/Caucasian
   02 African-American
   03 Hispanic-American
   04 Asian-American
   05 Other

[RECRUIT A MIX]
[DOCUMENT ON GRID; NOT A SCREENING CRITERION]

10. Determine Gender
    [RECRUIT A MIX]
    [DOCUMENT ON GRID; NOT A SCREENING CRITERION]
11. **[ASSESS AND VERIFY ABILITY TO SPEAK AND UNDERSTAND ENGLISH]**

Your interview will be held on ___________________ at ______________and will last for approximately 45 minutes. Because we know your time is valuable, at the end of the discussion we will pay you $XXX for participating.

Are you willing to attend?
Yes
No **[THANK & TERMINATE]**

Name____
Address____
City/State/Zip____
Day Number________________________**Night Number**_______________________

*IDIs, in-depth interviews.

Technical Appendix Section B

Worksheet: Antibiotic Selection

Please rank from 1-12. When antibiotic treatment is appropriate, how do the following impact the antibiotic that you choose to prescribe?

Factors for Antibiotic Selection	Influence on Antibiotic Selection
Illness severity	
Guidelines	
Cost	
Patient request	
Sample access	
Familiarity with dosing and outcome	
Patient allergies/medical history	
Patient compliance	
Concern for antibiotic resistance	
Ease of writing	
Pharmaceutical representatives	
Patient comorbidities	
Other	

1 = most influence
12 = less influence
City: ______________________ *Date:* ________________ *Time:* __________

Technical Appendix Section C

Clinical Vignettes

Family Practitioners

Cystitis

Case Scenario: You are in the Internal Medicine outpatient clinic seeing Ms. Brown, a 44-year-old female. She began to experience urgency leading to frequent urination and dysuria yesterday. Her urinalysis is positive for leukocyte esterase and reveals 20-40 white blood cells. She has no medication allergies and is otherwise healthy. You diagnose Ms. Brown with acute cystitis.

1. Which antibiotic would you prescribe and why (looking for nitrofurantoin, (macrodantin or macrobid), trimethoprim-sulfamethoxazole (Bactrim), Fosfomycin trometamol, Pivmecillinam)?
2. Data show that many of your peers choose broad-spectrum antibiotics like ciprofloxacin for this condition. Why do you think ciprofloxacin is frequently prescribed for this condition?

Internists

Sinusitis

Case Scenario: You are in the Internal Medicine outpatient clinic seeing Michelle, an otherwise healthy 36-year-old female, who complains of fever and increasing nasal discharge over the past 5 days after having initially recovered from a cold. She is concerned because her symptoms have been gradually getting worse. Physical exam reveals a temperature of 101.1°F, erythematous and enlarged nasal turbinates with purulent discharge on the right and tenderness over her right maxillary sinus. Her lung exam is clear. You decide that she most likely has acute bacterial rhinosinusitis.

1. Which antibiotic would you prescribe and why (looking for amoxicillin or amoxicillin-clavulanate (Augmentin)?
2. Data show that many of your peers, incorrectly, choose chose broad-spectrum antibiotics like azithromycin for this condition. Why do you think azithromycin is frequently prescribed for this condition?

NPs/PAs

Pharyngitis

Case Scenario: You are in the Pediatrics outpatient clinic seeing Luke, an otherwise healthy 9-year-old boy. His parents tell you that he has had severe sore throat for four days. He has fever, pharyngeal erythema, tonsillar exudate, and one enlarged anterior cervical lymph node on the right. You perform a rapid strep test and it's positive. The patient has group A streptococcal pharyngitis.

1. Which antibiotic would you prescribe and why (looking for penicillin or amoxicillin)?
2. Data show that many of your peers choose chose broad-spectrum antibiotics like azithromycin for this condition. Why do you think azithromycin is frequently prescribed for this condition?

Pediatricians

Otitis Media

Case Scenario: You are in the Pediatrics outpatient clinic seeing Rachel, an otherwise healthy 16-month-old girl. Her parents tell you that she has had fevers to 103°F and irritability, which started yesterday. She has been pulling at her right ear. Her parents are concerned that she has an ear infection and needs antibiotics. You've determined through a clinical exam

that the child has acute otitis media. Due to the patient's age and her certain diagnosis, you decide to prescribe an antibiotic.

1. Which antibiotic would you prescribe and why (looking for amoxicillin)?
2. Data show that many of your peers, incorrectly, choose chose broad-spectrum antibiotics like azithromycin for this condition. Why do you think azithromycin is frequently prescribed for this condition?
